# The Effect of Roasting on Oil Content, Fatty Acids, Bioactive Compounds and Mineral Contents of Purslane (*Portulaca oleracea* L.) Seeds

**DOI:** 10.3390/foods14050732

**Published:** 2025-02-21

**Authors:** Fahad Al Juhaimi, Zehra Beyza Atasoy, Nurhan Uslu, Mehmet Musa Özcan, Isam A. Mohamed Ahmed, Noman Walayat

**Affiliations:** 1 Department of Food Science & Nutrition, College of Food and Agricultural Sciences, King Saud University, Riyadh 11451, Saudi Arabia; faljuhaimi@ksu.edu.sa (F.A.J.); iali@ksu.edu.sa (I.A.M.A.); 2 Department of Food Engineering, Faculty of Agriculture, Selcuk University, 42079 Konya, Turkey; beyza_atasoy99@hotmail.com (Z.B.A.); nurhanuslu.gmuh@gmail.com (N.U.); 3 College of Tea Science and Tea Culture, Zhejiang Agriculture and Forestry University, Hangzhou 310007, China; noman.rai66@gmail.com

**Keywords:** purslane (*Portulaca oleracea*), seed, roasting, phenolic component, fatty acid, mineral

## Abstract

In this study, the effect of oven and microwave roasting at different times on oil content, total phenol, flavonoid, fatty acids, phenolic components and mineral contents of purslane seeds was investigated. The total phenolic quantities of the purslane seeds roasted in the oven and microwave were characterized to be between 252.0 ± 1.80 (180 °C/5 min in the oven) and 256.6 ± 3.51 (10 min in the oven), and between 216.3 ± 0.28 (720 W/15 min in the microwave) and 203.7 ± 1.93 GAE/100 g (30 min in the microwave), respectively. The highest total flavonoid (613.8 ± 4.36 mg QE/100 g) was detected in the application of roasting in the oven for 10 min. Roasting in the oven for 5 min caused a decrease in the total flavonoid content (584.3 ± 4.95 mg QE/100 g), while roasting for 10 min caused an increase in the flavonoid content (613.8 ± 4.36 mg QE/100 g). The oil yields of purslane seed samples roasted in the oven for 5 min and 10 min were defined as 40.40 ± 0.99% and 45.00 ± 0.71%, respectively. Statistical differences were observed between the oil, total phenol and flavonoid contents of the samples depending on the roasting times in the oven and microwave (*p* ≤ 0.01). The protein contents of the purslane seeds were established to be between 27.89 ± 0.279% (control) and 37.24 ± 0.407% (10 min in the oven). The calcium (Ca) contents of the purslane seeds changed between 8314.99 ± 327.53 ppm (5 min in the oven) and 4340.62 ± 498.45 ppm (15 min in the microwave), while the phosphorus contents varied between 4905.13 ± 43.02 ppm (15 min in the microwave) and 4051.23 ± 6.39 ppm (unroasted). In addition, the potassium content was found to be between 4565.89 ± 153.47 (5 min in the oven) and 3904.02 ± 7.17 ppm (unroasted). It was also observed that the purslane seeds roasted in the oven for 10 min maintained a linolenic fatty acid content of up to 65.57%. Considering the bioactive properties and phytochemical components of purslane seeds roasted in both roasting systems, they are important in terms of the nutritional enrichment of foods as a food supplement.

## 1. Introduction

Purslane (*Portulaca oleracea* L.), which has numerous horizontal stems and belongs to the Portulacaceae family, is an annual plant with a succulent structure and is branched with reddish stems [1,2]. Due to its salt tolerance, purslane is considered as a possible halophytic species for the desalination of saline soils and drainage water reuse systems [3,4]. Purslane plants can grow in tropical and subtropical countries of the world, and they grow widely in America, Asia and Europe [5,6]. Purslane oil has the highest amount of omega-3 fatty acids compared to other vegetables and is a potential nutrient due to its other biologically active compounds [5,7]. The total fatty acid contents of Australian purslane cultivars were found to be 1.5–2.5 mg/g in leaves on a fresh weight basis, 0.6–0.9 in stems and 80–170 mg/g in seeds [5]. Purslane contains glutathione, terpenoids, alkaloids, phenolic compounds (especially flavonoids) and volatile oils [8,9,10,11]. Purslane, known as the “vegetable for longevity” in Chinese culture, is an edible plant used as a diuretic, antipyretic, antiseptic and antispasmodic [12,13]. However, some pharmacological roles of purslane have been discovered, including antibacterial, analgesic, spasmolytic and wound healing [12,13]. The flavonoids and phenolic acids found in purslane improve inflammation and diabetes complications through their antioxidant activities [14]. Purslane has a variable chemical composition with various bioactive compounds and biological potential [15]. Purslane’s K, Ca, Mg, N, P and Fe contents were defined as 3.71 g/100 g, 2.39 g/100 g, 0.58 g/100 g, 2.17 g/100 g, 0.35 g/100 g and 0.0324 g/100 g, respectively [16]. Also, phenolic compounds and oleracein derivatives of extracts of purslane leaves have been reported to have antioxidant properties [17]. Flavonoids, a subgroup of phenolic constituents, have a wide range of mechanisms of action on plants, including antioxidant activity and increasing resistance to environmental stresses [18,19,20]. Roasting processes and conditions have been reported to cause significant physico-chemical changes in the color, aroma, fatty acid profile, phytochemicals and bioactive compounds of vegetable oils (edible seeds and nuts) [21]. Generally, when the components of oilseeds are taken into account, the temperature–time relationship varies between 180 and 220 °C according to a previous study [21], in which they applied different drying methods to purslane leaves and examined their moisture contents. According to this study, the moisture contents were recorded as 0.31%, 0.12% and 0.24% in the drying process carried out with vacuum, microwave and infrared methods, respectively [12]. The total phenolic contents of purslane samples collected from Antalya were recorded as 14.86 mg GAE/g, 5.22 mg GAE/g, 3.85 mg GAE/g, 5.44 mg GAE/g and 6.53 mg GAE/g in fresh, freezing, sun, vacuum oven and hot air oven drying methods, respectively [9]. The total phenolic contents of Mersin samples were determined as 17.95 mg GAE/g, 6.34 mg GAE/g, 6.73 mg GAE/g, 4.40 mg GAE/g, 9.32 mg GAE/g using fresh, freezing, sun, vacuum oven and hot air oven drying methods, respectively [9]. In general, most cultivated and edible wild plants are consumed through cooking. The changes in the bioactive components, polyphenol contents, fatty acids and mineral contents of purslane seeds during cooking constitute the importance of the study. Since the above-ground parts of the purslane plant are used in baked goods, pastries, salads and cooking, it is used with its seeds. Therefore, its seeds are beneficial for people when consumed in this way. The purpose of this study was to reveal the effect of oven and microwave roasting for different periods of time on the oil content, total phenol, flavonoid, fatty acids, phenolic component and mineral contents of purslane seeds.

## 2. Material and Methods

### 2.1. Material

Purslane seeds used in current study were provided from a commercial seed company in Konya in Türkiye. After the seeds were brought to the laboratory, they were roasted with two different roasting methods (oven and microwave), and the analyses specified in the Methods section were performed.

### 2.2. Methods

#### 2.2.1. Sample Preparation

**Figure 1:**
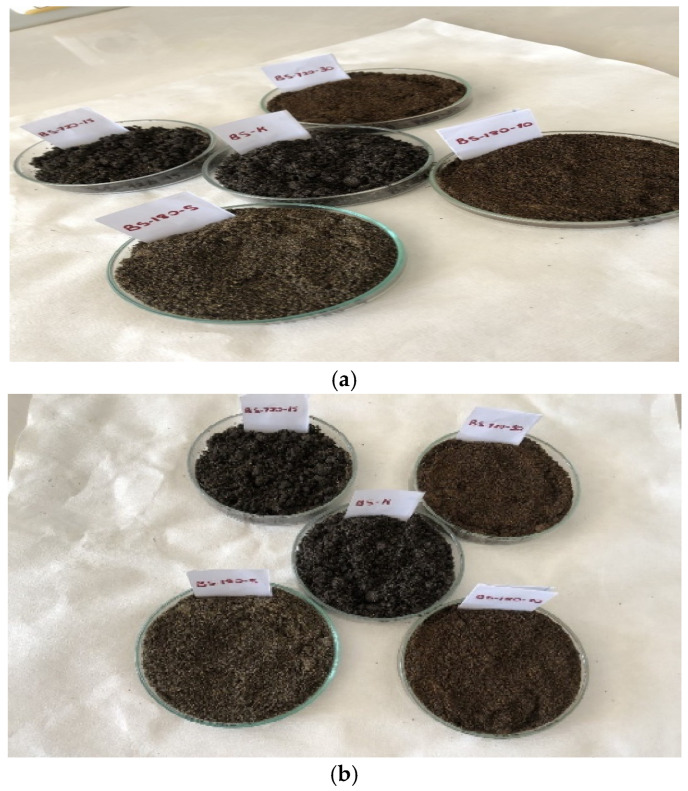
Purslane seeds subjected to roasting processes. (**a**) Oven roasting. (**b**) Microwave roasting.

The roasting process was carried out under controlled conditions in an oven (NÜVE FN O55, Ankara, Türkiye) at 180 °C for 5 and 10 min, and in a microwave oven (Arçelik MD 595, Istanbul, Türkiye) at 720 Watt for 15 and 30 min (Figure 1) [5]. These temperatures and times were chosen because oilseeds are generally roasted at these temperatures (±20–40 °C depending on seed size) and times. Purslane seeds were ground in a laboratory-type mini grinder (Mulino AE 151 ARZUM, Istanbul, Türkiye) to pass through a 0.5 mesh sieve.

#### 2.2.2. Preparation of Purslane Seed Extract

After purslane seeds roasted in the microwave (720 Watt for 15 and 30 min) and in the oven (180 °C for 5 and 10 min in the oven) for different time were ground, 5 g of purslane seeds was weighed into extraction tubes. Then, 10 mL of methanol/water (MeOH/H_2_O, 80:20, *v*/*v*) solution was added. It was kept in a shaking water bath at room temperature for 1 h. Then, it was centrifuged (Hermle Z-200A, Wehingen, Germany) at 6000 rpm for 10 min. The lower phase was taken into a flask and evaporated in a rotary evaporator (Heidolp, Schwabach, Germany) at 45 °C. The volume was made up to 10 mL with methanol/water (MeOH/H_2_O, 80:20, *v*/*v*) solution.

#### 2.2.3. Moisture Content

The moisture content of unroasted and roasted purslane seed samples were specified using Kern Dbs 60-3 (Balingen, Germany) infrared moisture analyzer.

#### 2.2.4. Total Flavonoid Amount

Total flavonoid contents of the samples were defined according to the methodology pointed out in previous study [21] and 5% NaNO_2_ (sodium nitrite) solution was added to all test tubes. After 5 min, 10% AlCl_3_ solution was added and, after 6 min, 2 mL NaOH solution was added. The absorbance was read at 510 nm. The results were defined as mg quercetin equivalents (QE)/100 g (dw).

#### 2.2.5. Total Phenolic Amount

Total phenol quantities of purslane samples were displayed using Folin–Ciocalteu reagent based on a previous study [22]. A total of 2.5 mL of Folin–Ciocalteu reagent and 2 mL of Na_2_CO_3_ solution were mixed onto 0.5 mL of extract. The total phenolic contents of the samples kept in the dark at room temperature for 2 h were determined at 725 nm wavelength in a spectrophotometer (Shimadzu, UV mini 1240, Kyoto, Japan). Findings were stated as mg GAE/100 g.

#### 2.2.6. Determination of Phenolic Compounds

For the identification of the phenolic component of purslane seeds that were not roasted, roasted in the oven at 180 °C/5 and 10 min and roasted in the microwave oven at 720 Watt/15 min and 720 Watt/30 min, 0.5 mL of the extracts filtered through 0.45 µm were transferred into vials. By adding 0.5 mL of methanol, the samples were made ready to be injected into the device. The phenolic component profile of the samples was determined at 280 nm using a HPLC (SCL-10 A VP-Shimadzu, Japan) device equipped with an Inertsil ODS-3 (5 µm; 4.6 mm × 250 mm) column and a PDA detector. The mobile phase was a mixture of 0.05% acetic acid in water (A) and acetonitrile (B) with a flow rate of 1 mL/min at 30 °C. The following elution program was employed: 0–0.10 min 8% B; 0.10–2 min 10% B; 2–27 min 30% B; 27–37 min 56% B; 37–37.10 min 8% B; and 37.10–45 min 8% B. It was recorded at 280 and 330 nm and the device was operated for 60 min for each sample. Samples were analyzed against standards on HPLC instrument (Shimadzu, Japan).

#### 2.2.7. Oil Extraction from Purslane Seeds

Ground purslane seeds (10 g) were weighed into a Soxhlet cartridge and placed in the extraction head. Petroleum ether was added to 2/3 of the Soxhlet flask and the extraction process started. Extraction was conducted at 50–60 °C for 5 h. After 5 h, the petroleum ether in the flask was removed via an evaporator. The weighing process continued until the oil amount of the samples reached a constant weight [23].

#### 2.2.8. Determination of Protein in Seeds

Protein amounts of purslane seeds were established according to the AOAC [24] method. Nitrogen percentage and protein were calculated using the following equation:

% Protein = Nitrogen × 6.25

#### 2.2.9. Mineral Contents

For mineral substance determination, approximately 0.2 g of powdered purslane seed samples was placed in burning containers containing 15 mL of pure HNO_3_ and 2 mL of H_2_O_2_ (30%; *w*/*v*). Purslane seed samples were burned in a microwave oven (Cem-MARS Xpress 6 One Touch Model, Atlanta, GA, USA, at 210 °C). After the combustion samples were filtered (number 42), the filtrates were collected in 50 mL bottles for elemental analysis in ICP-OES. Samples were analyzed against standards on ICP-OES instrument (Agilent- 5110, Santa Clara, CA, USA) [25].

**Working conditions of ICP-OES:** instrument: ICP-OES; RF Power: 0.7–1.5 kw (1.2–1.3 kw for Axial); plasma gas flow rate (Ar): 10.5–15 L/min. (radial) 15 “(Axial); auxiliary gas flow rate (Ar): 1.5”; viewing height: 5–12 mm; copy and reading time: 1–5 s (max. 60 s); copy time: 3 s (max. 100 s).

#### 2.2.10. Fatty Acid Composition

Oil samples were converted to fatty acid methyl esters (FAME). For the esterification process. First, 10 mL n-Hexane (C_6_H_14_) was added to 0.1 g of oil sample and dissolved, and then it was centrifuged (Hermle Z-200A, Germany) at 4500 rpm for 30 min. Then, 2 N methanol KOH was added and the solution was mixed using vortex for a short time and then kept in the dark at room temperature for 30 min. After the phase separation process was completed, 1 mL of the supernatant was taken and transferred to vials and made ready for analysis. Methyl esters of fatty acids were detected in a gas chromatography device (Shimadzu GC-2010, Kyoto, Japan) [26]. Sigma brand 99.92% purity fatty acid methyl esters were used as standards.

Gas chromatography operating conditions:

Detector: Flame Ionization Detector (FID);

Capillary column: Teknokroma TR CN100, P/N TR 882, 162 fused silica column;

Detector and injection block temperature: 260 °C;

Carrier gas: N_2;_

Flow rate: 1.51 mL/min;

Split ratio: 1/40 mL/min;

Injection block total flow rate: 80 mL/min.

### 2.3. Statistical Analysis

After the results were subjected to variance analysis using the Minitab 16 statistical program(Windows 10), the statistically significant means were compared with the Tukey multiple comparison test. The results are given with their mean values (*n* = 3) and standard deviations.

## 3. Results and Discussion

### 3.1. Moisture and Bioactive Properties of Purslane Seeds

Two different roasting methods were applied to purslane seed samples at different times and temperatures, and the contents of moisture, total phenolic, total flavonoid and oil are presented in Table 1. The highest moisture content of purslane seeds was found to be 5.84 ± 0.21% in the untreated seeds, whereas that of seeds roasted in the oven for 5 and 10 min was 3.04 ± 0.34% and 2.18 ± 0.45%, respectively. The moisture quantities of purslane seeds roasted in the microwave were specified to be between 2.18 ± 0.04 (30 min) and 5.84 ± 0.21% (Control). The moisture quantity of purslane seeds roasted in the microwave for 30 min was lower than those roasted for 15 min. Statistical differences were observed between the oil, total phenol and flavonoid contents of the samples depending on the roasting times in the oven and microwave (*p* ≤ 0.01). The experimental results are in accordance with Hosseini et al. and Stroescu et al. [27,28]. The total phenolic amounts of purslane seed samples were characterized to be between 203.7 ± 1.93 (30 min in microwave oven) and 262.5 ± 1.92 mg GAE/100 g (control). The total phenolic contents of purslane seeds roasted in the oven for 5 and 10 min were 252.0 ± 1.80 and 256.6 ± 3.51 mg GAE/100 g, respectively. The total amount of phenolic in the samples processed in the microwave oven for 15 min was recorded as 216.3 ± 0.28 mg GAE/100 g, while that of seeds roasted for 30 min was 203.7 ± 1.93 mg GAE/100 g. The total phenolic and flavonoid contents of the samples roasted in the oven were higher than those roasted in the microwave. The increase in the total phenol content may have been due to an increased release due to cell lysis during extraction. The total phenolic contents of purslane samples collected from Antalya were recorded as 14.86 mg GAE/g, 5.22 mg GAE/g, 3.85 mg GAE/g, 5.44 mg GAE/g and 6.53 mg GAE/g using fresh, freezing, sun, vacuum oven and hot air oven drying methods, respectively [9]. The total phenolic contents of Mersin samples were determined as 17.95 mg GAE/g, 6.34 mg GAE/g, 6.73 mg GAE/g, 4.40 mg GAE/g and 9.32 mg GAE/g in fresh, freezing, sun, vacuum oven and hot air oven drying methods, respectively [9]. Roasting methods (oven and microwave) applied to purslane seeds caused a decrease in the total phenolic substance content. The total phenol content of ungerminated purslane seeds increased during 3 days of germination (466.72 μg/g) compared to ungerminated seeds (315.02 μg/g) [29]. Mousavi et al. determined 121.09 mg GAE/kg total phenol in the methanol extract of purslane seed [30]. In other study, the total phenolic content extracted from purslane seed was recorded as 62.12 mg GAE/kg [31]. Binici et al. examined the effect of different drying methods on the nutritional composition of purslane, and the total phenolic amounts of the drying methods applied to the purslane plant collected from the Antalya region in the sun, in the vacuum oven and in the hot air oven were determined as 5.22, 3.85, 5.44 and 6.53 mg GAE/g, respectively [32]. The total flavonoid quantity of purslane seed is depicted in Table 1. The highest total flavonoid content (613.8 ± 4.36 mg QE/100 g) was detected in the application of roasting in the oven for 10 min. The lowest total flavonoid amount in a purslane sample was 458.6 ± 4.95 mg QE/100 g. Roasting in the oven for 5 min caused a decrease in the total flavonoid content (584.3 ± 4.95 mg QE/100 g), while roasting for 10 min caused an increase in the flavonoid content (613.8 ± 4.36 mg QE/100 g). In the microwave roasting process, a decrease was observed in the total flavonoid content of purslane seeds. The lowest oil yield was established in the control group, with 38.85 ± 2.05%. Meanwhile, the oil yield was determined as 40.40 ± 0.99% and 45.0 ± 0.71% after roasting in the oven for 5 and 10 min, respectively, and the oil yield was determined as 42.80 ± 0.85% and 45.50 ± 1.27% as a result of roasting purslane seeds in the microwave for 15 and 30 min, respectively. In all the roasting processes applied to purslane seeds, a higher oil yield was obtained compared to the control group. The reason for this may be that the applied temperature accelerates the movement of molecules, making the lipid layers of cell membranes more fluid [33,34,35,36,37]. The roasting process we applied to purslane seeds was similar to a study conducted by previous researchers [38]. However, our results were higher than those of previous studies [37,39,40]. These differences may be due to differences such as the solvents used, roasting temperatures and some analytical conditions.

### 3.2. Phenolic Compounds of Roasted and Unroasted Purslane Seeds

The phenolic component results for unroasted purslane seeds and the oven roasting and microwave roasting processes are depicted in Table 2. According to the phenolic component results, the most abundant phenolic components in purslane seeds were kaempferol, catechin and cinnamic acid. The amount of kaempferol in purslane seeds roasted in the oven and roasted in the microwave varied between 5.40 ± 0.68 (microwave/30 min) and 6.09 ± 0.24 mg/100 g (microwave/15 min), and it was the component with the highest amount. The amount of catechin in the samples was 1.84 ± 0.05 (oven/5 min) and 4.15 ± 1.32 mg/100 g (control). According to the phenolic component determination results, the amounts of *p*-coumaric acid were found to be between 0.05 ± 0.04 (microwave/30 min) and 0.38 ± 0.29 mg/100 g (control). The greatest amount of 3,4-dihydroxybenzoic acid was found in the purslane seeds treated in the oven for 10 min (0.89 ± 0.84 mg/100 g), followed by the control group (0.74 ± 0.61 mg/100 g) and the seeds roasted in the microwave for 30 min (0.60 ± 0.50 mg/100 g). While the gallic acid amount of the samples treated for 5 min in the oven was 0.83 ± 0.21 mg/100 g, the amount of gallic acid in the samples treated for 10 min was determined as 1.13 ± 0.67 mg/100 g. The gallic acid amount of purslane seeds roasted for 15 min in the microwave was determined as 1.00 ± 0.69 mg/100 g, and the gallic acid amount of seeds roasted for 30 min was determined as 1.49 ± 0.71 mg/100 g. According to the analysis results, the rutin amounts were established to be between 0.17 ± 0.06 (control) and 0.36 ± 0.33 mg/100 g (oven/5 min), while quercetin amounts were defined as 0.21 ± 0.10 (oven/5 min) and 3.34 ± 4.94 (oven/10 min). The caffeic acid contents of oven-roasted and microwave-roasted purslane seeds were found to be between 0.01 ± 0.01 (10 min) and 0.08 ± 0.06 (control), and between 2.35 ± 0.63 (15 min) and 4.15 ± 1.32 mg/100 g (control), respectively. In addition, the syringic acid contents of oven-roasted seeds were found to range between 0.07 ± 0.02 (10 min) and 0.44 ± 0.18 (control), while the syringic acid contents of microwave-roasted purslane seeds were found to be between 0.03 ± 0.01 (30 min) and 0.44 ± 0.18 Mg/100 g (control). Also, ferulic acid amounts in oven- and microwave-roasted purslane seeds were found to be between 0.04 ± 0.03 (control) and 0.12 ± 0.08 mg/100 g (10 min), and between 0.04 ± 0.03 (control) and 0.07 ± 0.05 mg/100 g (30 min), respectively. The resveratrol contents of oven-roasted purslane seeds were determined to be between 0.02 ± 0.01 (5 min) and 0.44 ± 0.02 (10 min), while the resveratrol contents of purslane seeds roasted in the microwave for 15 and 30 min were between 0.01 ± 0.00 (15 min) and 0.03 ± 0.01 (control and 30 min). Although the phenolic compound values of purslane seeds roasted at different roasting types and times showed partial differences, no statistically significant difference was found between the compound amounts (*p* > 0.05). The fluctuations in the amounts of phenolic compounds depending on the roasting time may have resulted from the interaction between the phenolic compounds of purslane seed and other components and biochemical reactions. Cinnamic acid is one of the phenolic components found in high amounts in purslane seed, and it was determined as 3.41 ± 1.05 mg/100 g during the roasting process in the oven for 10 min. The phenolic component amounts of purslane seed showed some differences with the results of previous studies [41,42]. Changes in the phenolic compound amounts of purslane seeds may have affected the release of phenolic compounds in the seed due to different roasting types, temperatures and durations.

### 3.3. Protein and Mineral Contents of Roasted and Unroasted Purslane Seeds

The protein amounts of raw and roasted purslane seeds varied between 10.08 ± 2.02% and 37.24 ± 0.407% (Table 3). The highest protein content was detected in the purslane seed sample that was roasted in the oven for 5 min, followed by the control group, with 27.89 ± 0.279%, and the sample that was roasted in the oven for 10 min, with 20.25 ± 0.783%. While the amount of protein in the samples roasted for 15 min in the microwave oven was determined as 11.25 ± 0.075%, the protein content was determined to be 10.08 ± 2.02% in the samples roasted for 30 min in the microwave oven. As the duration of the microwave roasting process increased, the protein amount in the purslane seed samples decreased. This may be due to the denaturation of the molecular structure of proteins with heating. The protein content of dried purslane seeds was established as 27.58% [43]. The protein amounts in the Egyptian variety were recorded as 23.7% (April 30), 27.1% (May 12), 23.6% (April 30) and 24.1% (May 12) [44]. The protein analysis results for the seeds were similar to most of the findings in the literature. The differences in protein content according to literature data may be due to damage of the protein caused by roasting temperatures.

The highest amount of any mineral substance was found for nitrogen, followed by calcium, phosphorus, potassium and magnesium. The amounts of nitrogen were specified to be between 16,126.91 ± 322.86 ppm (microwave for 30 min) and 59,583.06 ± 650.92 ppm (oven for 5 min). The highest amount of calcium was recorded as 8314.99 ± 327.53 ppm when roasted in the oven for 5 min, while the lowest amount of calcium was assessed as 4340.62 ± 498.45 ppm when roasted in the microwave for 15 min. The amount of potassium was determined as 4565.89 ± 153.47 ppm (oven for 5 min), 4217.67 ± 86.43 ppm (microwave for 30 min), 4163.45 ± 187.49 ppm (microwave for 15 min) and 4140.41 ± 66.07 ppm (oven for 10 min). The P contents of purslane seeds roasted in the oven and microwave were determined to range from 4051.23 ± 6.39 (control) to 4853.37 ± 102.7 mg/kg (10 min) and from 4051.23 ± 6.39 (control) to 4905.13 ± 43.0 2 mg/kg (10 min), respectively. In addition, the S contents of purslane seeds roasted in oven for 5 to 10 min were determined to range from 2390.60 ± 35.71 (control) to 2594.18 ± 19.86 mg/kg (10 min), while the S contents of purslane seeds roasted in microwave for 15 to 30 min were determined to range from 2238.31 ± 187.57 (15 min) to 2552.26 ± 86.09 mg/kg (30 min). In addition, iron, copper, zinc, manganese and boron minerals were detected in lower amounts. The maximum iron mineral was recorded as 169.34 ± 26.74 ppm (oven for 5 min). Also, the highest zinc content was found to be 56.31 ± 0.640 ppm as a result of roasting in the oven for 10 min, while the lowest amount was found in the control group, with 50.06 ± 0.136 ppm. The amount of copper was found to be between 17.47 ± 1.19 ppm and 21.51 ± 1.24 ppm, and the manganese content was determined to be between 25.04 ± 1.55 ppm and 39.71 ± 1.04 ppm. The least amount of mineral substance in purslane seed samples was found for boron, with 8.79 ± 1.31 ppm. Differences in the amounts of macronutrients, such as Ca and P, and micronutrients, such as Fe and Zn, in the seeds due to different roasting types, temperatures and durations may be due to thermal decomposition, oxidation or other factors such as seed size and shell thickness.

The differences between the macro and micro element contents of purslane seeds roasted at different roasting types and times were found to be statistically significant (*p* < 0.05). Alam et al. examined the physiological properties and mineral contents of various purslane collected from different regions of Malaysia [45].

### 3.4. Fatty Acid Compositions of the Oils Extracted from Unroasted and Roasted Purslene Seeds

**Figure 2:**
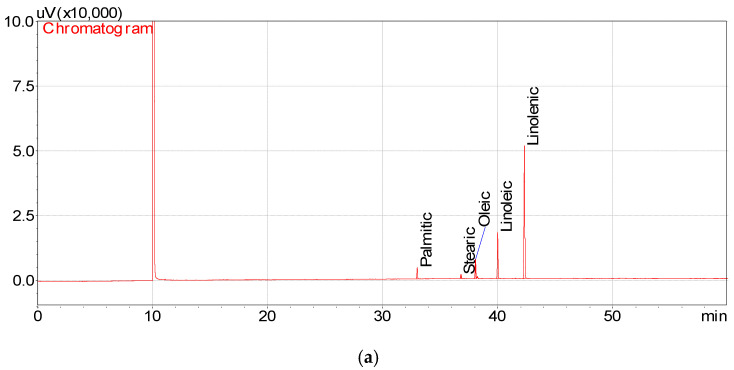

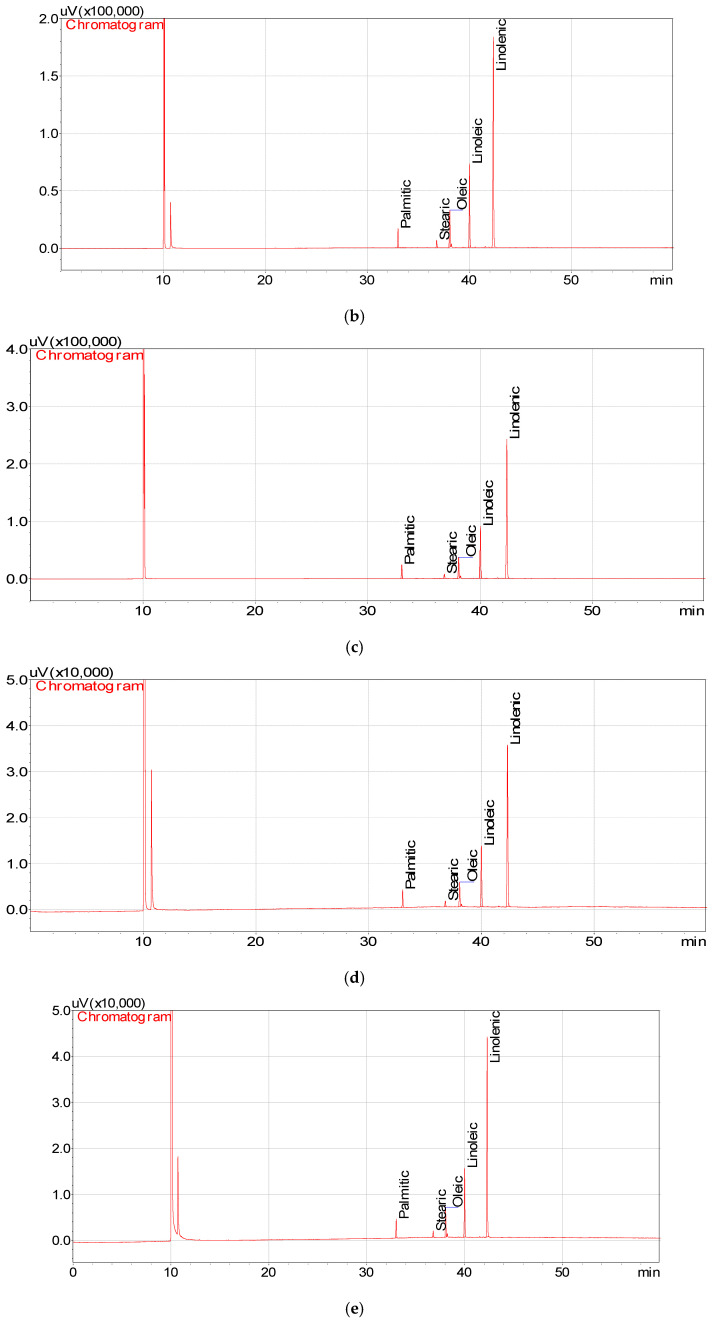
Fatty acid chromatogram of the oils extracted from roasted purslane seeds: (**a**) Control; (**b**) purslane seed oil (oven at 180 °C for 5 min); (**c**) purslane seed oil (oven at 180 °C for 10 min); (**d**) purslane seed oil (microwave oven at 720 Watt for 15 min); (**e**) purslane seed oil (microwave oven at 720 Watt for 30 min).

The fatty acid compositions of purslane seed oils are stated in Table 4 and Figure 2 Linolenic acid was the fatty acid with the highest amount, with 64.49 ± 0.11% (control), 62.49 ± 0.19% (in the oven/5 min), 65.57 ± 0.28% (in the oven/10 min), 62.90 ± 0.92% (microwave/15 min) and 64.87 ± 0.96% (microwave/30 min). The most abundant fatty acid after linolenic acid was linoleic acid, and it was determined as 21.04 ± 0.00% (control), 21.98 ± 0.10% (in the oven for 5 min), 20.72 ± 0.03% (in the oven for 10 min), 22.32 ± 0.37% (microwave for 15 min) and 21.00 ± 0.22% (microwave for 30 min). According to the analysis results, the amount of oleic acid was identified to be between 7.78 ± 0.02% and 9.15 ± 0.08%. In addition, the palmitic acid amount changed between 4.41 ± 0.29% (in the oven for 10 min) and 5.43 ± 0.24% (in the microwave for 15 min). According to our analysis results, the least abundant fatty acid in purslane seed oil is stearic acid, with amounts varying between 1.49 ± 0.02% and 1.74% ± 0.05. The palmitic acid content of oils obtained from purslane seeds roasted in the oven was found to be higher than that of microwave roasted seeds, while the stearic acid content was found to be relatively low. In addition, the linoleic acid content of oils extracted from microwave roasted seeds was found to be relatively higher than that of oven roasted. This situation is thought to be due to the high oxidation effect of oven temperature on linoleic acid.

The differences in oleic acid contents of oils extracted from purslane seeds roasted in the oven for different times and the differences in oleic acid contents of oils extracted from seeds roasted in the oven and microwave were found to be statistically significant (*p* ≤ 0.01). Purslane seed oil contains a palmitic acid (16:0) amount of 164.3 g/kg, a stearic acid amount of 30.9 g/kg, an oleic acid amount of 163.6 g/kg, a linoleic acid (18:2) amount of 336.3 g/kg and a α-linolenic acid (C18:3ω3) amount of 267.7 g/kg [46].

## 4. Conclusions

The oil content, total phenol and flavonoid contents of purslane seeds roasted in the oven for 10 min were higher than those roasted for 5 min. In addition, an increase in the oil, total phenol and flavonoid contents of purslane seeds was observed in the sample roasted in the microwave for 15 min. As a result of roasting in the oven at 180 °C for 5 min, an increase in palmitic, oleic and linoleic acids was found and a decrease in stearic and linolenic acids was detected. Purslane seeds contain sodium, calcium, potassium and iron, which play an important role in muscle and bone development. Purslane seeds, which have a rich omega-3 fatty acid content, continue to attract consumers’ attention every day. It acts as a good antioxidant thanks to the phenolic components, such as kaempferol and catechin, in its structure. According to our study results, purslane seeds roasted in the oven at 180 °C for 5 min caused an increase in both the amount of protein and minerals, and the amount of fatty acids such as palmitic, oleic and linoleic acid. It is recommended to consume purslane seeds with water, milk and yogurt after roasting and grinding due to their benefits in providing biologically active substances and compounds necessary for human nutrition. In addition, the healthy fatty acid content of purslane seed oil increases its potential to be a new nutritious food source day by day. Future studies will examine the changes in bioactive components, phenolic compounds and fatty acid amounts as a result of germination of purslane seeds, of which the aerial part can be consumed. In addition, changes in the phytochemical components and nutrient contents of purslane seeds will be investigated by preserving them in cold environments with different roasting techniques.

## Figures and Tables

**Table 1:** Effects of different roasting methods on the moisture, total phenolic components, total flavonoid and oil contents of the purslane seed (dry weight (dw)).

Process	Times	Moisture Contents (%)	Oil Contents (%)	Total Phenolic Contents (mg GAE/100 g)	Total Flavonoid Contents (mg QE/100 g)
Oven	Control	5.84 ± 0.21 ^A^*	38.85 ± 2.05 ^D^	262.5 ± 1.93 ^A^	610.0 ± 17.84 ^A^
	5 min	3.04 ± 0.34 ^B^	40.40 ± 0.99 ^C^	252.0 ± 1.80 ^AB^	584.3 ± 4.95 ^C^
	10 min	2.18 ± 0.45 ^C^	45.00 ± 0.71 ^A^	256.6 ± 3.51 ^B^	613.8 ± 4.36 ^B^
Microwave	15 min	2.40 ± 0.22 ^C^	42.80 ± 0.85 ^B^	216.3 ± 0.28 ^C^	489.0 ± 5.95 ^D^
	30 min	2.18 ± 0.04 ^C^	45.50 ± 1.27 ^A^	203.7 ± 1.93 ^D^	458.6 ± 4.95 ^E^

* ^A–E^: Data represent n = 3 ± standard deviation. The values stated in the same row were found to be statistically significant (*p* ≤ 0.01).

**Table 2:** Phenolic components of purslane seeds with different roasting methods (mg/100 g (dry weight (dw))).

Methods	Times	Gallic	3,4-Dihydroxybenzoic	Catechin	Caffeic	Syringic	Rutin	*p*-Coumaric	Ferulic	Resveratrol	Quercetin	Cinnamic	Kaempferol
Oven	Control	1.06 ± 0.14 *	0.74 ± 0.61	4.15 ± 1.32	0.08 ± 0.06	0.44 ± 0.18	0.17 ± 0.06	0.38 ± 0.29	0.04 ± 0.03	0.03 ± 0.01	0.31 ± 0.12	2.96 ± 0.47	5.74 ± 0.38
	5 min	0.83 ± 0.21	0.12 ± 0.01	1.84 ± 0.05	0.03 ± 0.01	0.17 ± 0.08	0.36 ± 0.33	0.18 ± 0.12	0.05 ± 0.02	0.02 ± 0.01	0.21 ± 0.10	2.63 ± 1.28	5.88 ± 0.33
	10 min	1.13 ± 0.67	0.89 ± 0.04	3.60 ± 1.6	0.01 ± 0.00	0.07 ± 0.02	0.24 ± 0.19	0.15 ± 0.12	0.12 ± 0.08	0.44 ± 0.02	3.34 ± 1.94	3.41 ± 1.05	5.49 ± 0.34
Microwave	Control	1.06 ± 0.14	0.74 ± 0.61	4.15 ± 1.32	0.08 ± 0.06	0.44 ± 0.18	0.17 ± 0.06	0.38 ± 0.29	0.04 ± 0.03	0.03 ± 0.01	0.31 ± 0.12	2.96 ± 0.47	5.74 ± 0.38
	15 min	1.00 ± 0.69	0.53 ± 0.02	2.35 ± 0.63	0.05 ± 0.03	0.08 ± 0.04	0.22 ± 0.03	0.14 ± 0.04	0.06 ± 0.01	0.01 ± 0.00	0.44 ± 0.04	1.30 ± 0.72	6.09 ± 0.24
	30 min	1.49 ± 0.71	0.60 ± 0.50	3.66 ± 1.70	0.04 ± 0.02	0.03 ± 0.01	0.22 ± 0.14	0.05 ± 0.03	0.07 ± 0.05	0.03 ± 0.00	1.03 ± 0.32	3.02 ± 0.82	5.40 ± 0.68

* The values stated in the same column were not found to be statistically significant (*p* > 0.05) (*n* = 3).

**Table 3:** Protein (%), macro and micro element contents (ppm (dry weight (dw))) of purslane seeds subjected to different roasting processes.

Methods	Times	Protein	N	P	K	Ca	Mg	S
Oven	Control	27.89 ± 0.279 ^b^*	44,623.91 ± 446.44 ^b^	4051.23 ± 6.39 ^d^	3904.02 ± 7.17 ^c^	5901.53 ± 415.47 ^bc^	2741.53 ± 4.12 ^c^	2390.60 ± 35.71 ^bc^
	5 min	37.24 ± 0.407 ^a^	59,583.06 ± 650.92 ^a^	4489.69 ± 143.49 ^c^	4565.89 ± 153.47 ^a^	8314.99 ± 327.53 ^a^	3257.20 ± 57.42 ^a^	2493.74 ± 68.68 ^ab^
	10 min	20.25 ± 0.783 ^c^	32,395.26 ± 125.17 ^c^	4853.37 ± 102.75 ^ab^	4140.41 ± 66.07 ^b^	4811.13 ± 243.82 ^cd^	2903.47 ± 39.14 ^bc^	2594.18 ± 19.86 ^a^
Microwave	15 min	11.25 ± 0.075 ^c^	17,993.15 ± 120.33 ^d^	4905.13 ± 43.02 ^a^	4163.45 ± 187.49 ^b^	4340.62 ± 498.45 ^d^	3017.87 ± 181.03 ^b^	2238.31 ± 187.57 ^c^
	30 min	10.08 ± 2.02 ^c^	16,126.91 ± 322.86 ^d^	4707.94 ± 113.71 ^b^	4217.67 ± 86.43 ^b^	6205.61 ± 124.07 ^b^	3019.43 ± 43.46 ^b^	2552.26 ± 86.09 ^ab^
**Methods**	**Times**	**Fe**	**Zn**	**Cu**	**Mn**	**B**		
Control	-	123.43 ± 14.88 ^bc^*	50.06 ± 0.136 ^b^	19.31 ± 0.391 ^b^	27.26 ± 0.338 ^cd^	4.85 ± 0.345 ^b^		
Oven	5 min	169.34 ± 26.74 ^a^	55.38 ± 1.69 ^a^	21.51 ± 1.24 ^a^	39.71 ± 1.04 ^a^	5.61 ± 0.083 ^b^		
	10 min	147.70 ± 12.23 ^ab^	56.31 ± 0.640 ^a^	20.78 ± 0.657 ^ab^	25.04 ± 1.55 ^d^	5.92 ± 0.071 ^b^		
Microwave	15 min	113.45 ± 1.23 ^c^	51.10 ± 2.97 ^b^	17.47 ± 1.19 ^c^	34.67 ± 3.35 ^b^	8.79 ± 1.31 ^a^		
	30 min	134.30 ± 3.80 ^bc^	55.78 ± 1.32 ^a^	20.74 ± 1.16 ^ab^	29.28 ± 1.13 ^c^	5.77 ± 0.397 ^b^		

* Data were calculated as the average of samples obtained over three replicates; ± indicates standard deviation. Values marked with different letters (^a–d^) in the same column were found to be statistically significant (*p* < 0.05).

**Table 4:** Fatty acid compositions of roasted and unroasted purslane seed oils (%).

Methods	Times	Palmitic	Stearic	Oleic	Linoleic	Linolenic
Oven	Control	4.47 ± 0.17 *	1.69 ± 0.04	8.31 ± 0.02 ^B^**	21.04 ± 0.00	64.49 ± 0.11
	5 min	4.65 ± 0.42	1.74 ± 0.05	9.15 ± 0.08 ^A^	21.98 ± 0.10	62.49 ± 0.19
	10 min	4.41 ± 0.29	1.53 ± 0.013	7.78 ± 0.02 ^C^	20.72 ± 0.03	65.57 ± 0.28
Microwave	Control	4.47 ± 0.17	1.69 ± 0.04	8.32 ± 0.02 ^B^	21.04 ± 0.00	64.49 ± 0.11
	15 min	5.43 ± 0.24	1.59 ± 0.13	8.52 ± 0.14 ^B^	22.32 ± 0.37	62.90 ± 0.92
	30 min	4.85 ± 0.13	1.49 ± 0.02	8.52 ± 0.02 ^B^	21.00 ± 0.22	64.87 ± 0.96

* Mean values without letters are not statistically significant (*p* > 0.05). ** (^A–C^) Data represent n = 3 ± standard deviation. The values stated in the same row were found to be statistically significant (*p* ≤ 0.01).

## Data Availability

The original contributions presented in this study are included in the article. Further inquiries can be directed to the corresponding author.
